# Cortical potential imaging using L-curve and GCV method to choose the regularisation parameter

**DOI:** 10.1186/1753-4631-4-S1-S4

**Published:** 2010-06-03

**Authors:** Narayan P Subramaniyam, Outi RM Väisänen, Katrina E Wendel, Jaakko AV Malmivuo

**Affiliations:** 1 Department of Biomedical Engineering, Tampere University of Technology, Tampere, Finland

## Abstract

**Background:**

The electroencephalography (EEG) is an attractive and a simple technique to measure the brain activity. It is attractive due its excellent temporal resolution and simple due to its non-invasiveness and sensor design. However, the spatial resolution of EEG is reduced due to the low conducting skull. In this paper, we compute the potential distribution over the closed surface covering the brain (cortex) from the EEG scalp potential. We compare two methods – L-curve and generalised cross validation (GCV) used to obtain the regularisation parameter and also investigate the feasibility in applying such techniques to N170 component of the visually evoked potential (VEP) data.

**Methods:**

Using the image data set of the visible human man (VHM), a finite difference method (FDM) model of the head was constructed. The EEG dataset (256-channel) used was the N170 component of the VEP. A forward transfer matrix relating the cortical potential to the scalp potential was obtained. Using Tikhonov regularisation, the potential distribution over the cortex was obtained.

**Results:**

The cortical potential distribution for three subjects was solved using both L-curve and GCV method. A total of 18 cortical potential distributions were obtained (3 subjects with three stimuli each – fearful face, neutral face, control objects).

**Conclusions:**

The GCV method is a more robust method compared to L-curve to find the optimal regularisation parameter. Cortical potential imaging is a reliable method to obtain the potential distribution over cortex for VEP data.

## Introduction

The electro- and magneto-encephalography (EEG, MEG) are the tools that are commonly used to record the brain activity on a sub millisecond time scale [1]. One of the important goals of EEG/MEG research is the localisation of the underlying sources giving rise to the activities on the scalp [2]. Such a localisation is not possible using the scalp EEG/MEG data alone. This stems from the theory of classical electromagnetic inverse problems, which says that different source configurations can give rise to same electric potential and magnetic field on the scalp [3].

The non-uniqueness in the inverse problem can be solved by determining the potential distribution over a closed surface, which encloses all the sources in the brain [4]. Strictly speaking, this is not an inverse solution as no information about the generators is obtained. Also, the same potential distribution over the cortex can be explained by more than one configuration of generators. The advantages of computing cortical potential distribution are a) It can be determined uniquely as solution space is restricted to a closed surface [4] and b) It might provide better and enhanced picture of what is going on inside the brain with significant hints about the suppositional sources. This is a spatial enhancement technique and the scalp potential is de-blurred and computed over the cortex. There exist many spatial enhancement algorithms [5-7]. In cortical potential imaging technique a volume conductor model of the head is constructed (realistic or spherical). The properties of the region between the cortex and scalp are only considered, which has no sources [8] and the Laplace’s equation is solved.

The tissue conductivity is an important issue in volume conductor modeling. Over the years, different values for tissue conductivity have been reported for head model. A wide range of values for the conductivity of skull has been reported. In their seminal article, Rush and Driscoll reported that the resistivity of skull was 80 times that of brain [9]. For a very long time, this value was used in modeling studies. In the year 2000 Ooestendorp *et al*. [10] reported a new value for resistivity ratio and it was 1:15:1 for brain:skull:scalp. Recent values for skull resistivity are reported in [11-13]. It is worth noting that, the accuracy of the inverse solution is affected by the value chosen for skull resistivity. Also, skull contains sinuses and areas of thinning. This impacts the volume current distribution and makes the current path longer in the areas which has holes. This makes localisation difficult [14].

The EEG inverse problem is ill-posed due to the fact that the transfer matrix, which relates the sources to measurements, is ill-conditioned. One way to deal with the ill-posedness is to apply regularisation techniques. There exist many regularisation techniques, the primary ones being Tikhonov regularisation and truncated singular value decomposition (TSVD). A key issue in the application of such regularisation methods is the selection of the optimal regularisation parameter. A detailed review about these methods can be found in [15].

Cortical potential distribution is of clinical importance and copious research has been done to enhance the spatial details of EEG [5-8]. By looking at the cortical potential distribution, a surgeon can do a better pre-surgical planning for e.g. in case of epileptic surgery [16]. Such information is also valuable to psychologists in the field of cognitive neuroscience. The VEP distribution over the cortex, for instance can provide better hints than scalp potential about the parts of the cortex which are more active for a certain external visual stimuli (faces, objects etc) [17].

The purpose of this study was to apply cortical potential imaging technique to a realistic head model constructed using VHM dataset and compare the L-curve and GCV method used to find the regularisation parameter. We used a real set of EEG data to demonstrate the effect of choosing regularization parameters using these two methods on the potential distribution over cortex.

## Methods

### Image modification

A realistic FDM model of the human head was constructed using VHM dataset, which excluded the brain and had cortex defined as closed surface. The same model was used for all the three subjects, without any modifications made taking the anatomical features of the subjects. The brain can be excluded based on Gauss’s law since the sources exist only inside the brain.

**Figure 1:**
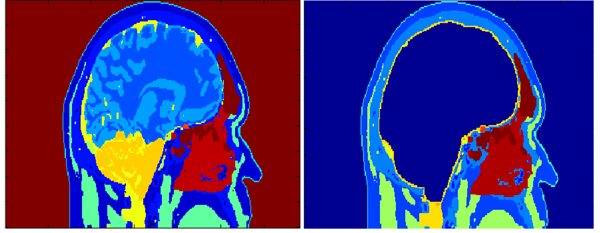
The original, presegmented VHM image is shown on the left. The modified VHM image after the exclusion of brain with CSF continuously surrounding the empty space is shown on the right.

The resolution of the presegmented, VHM template image dataset was 1mm X 1mm X 1mm. The first step was to exclude the brain from the original images and define a closed surface comprising of cerebrospinal fluid (CSF). A mask was defined, which had the tissue type – white matter (WM), grey matter (GM), CSF, neural tissue defined as zero and the rest as one. This mask was dilated in all the three dimensions using morphological operation. This dilated mask was then multiplied element-by-element with the original image. In doing so, the brain was excluded from the model and the CSF covered the empty space inside. The original image and the modified images are shown in Figure 1.

### Tissue conductivity

The tissue conductivities for this study were referred from the literature [10-13]. The ratio of 1:15:1 is assumed for scalp:skull:brain resistivity. The conductivities are assumed to be isotropic. Table 1 gives the list of tissues included in the model and their respective conductivity values.

**Table 1:** Tissue Conductivities

Tissue Type	Conductivity (S/m)
Empty	0.0015
Fat	0.04
Skin	0.43
Eye	0.33
Skeletal Muscle	0.1
Blood	0.625
CSF	1.53
Skull	0.028
Connective Tissue	0.04
Internal Air	0.002

Conductivity values used in the volume conductor model. All the values were referred from [12],[13] and [14].

### Forward transfer matrix

The FDM model consisted of 103799 cortical nodes. The cortical nodes were defined as the nodes lying at the interface of CSF and the empty space. In order to find a relation between cortical potential and the scalp potential, a forward transfer matrix was obtained. Since the number of cortical nodes is high, which would result in higher computational time, the nodes were grouped into equal sized source areas. In order to achieve this, first a sphere was divided into *n* equal sized source areas. This sphere was placed at the origin of the cortex and these areas were projected on the surface of cortex.

Superposition principle was used to obtain the forward transfer matrix. One source area at a time was made active (i.e. setting one source area to 1 volt), and the rest were set to zero [18]. The resulting potential at electrode locations on scalp was found. This information forms one column of the transfer matrix. This procedure was repeated for the rest of the source areas, resulting in a matrix of size *m* x *n*, where m is the number of electrode locations and n, the number of source areas. The relationship between the number of source areas and number of reconstructable basis vectors for different noise levels has been investigated by Ryynänen et al [19] for spherical head model. Based on this study, the cortex was divided into 2000 equal sized source areas.

### Actual EEG potentials

The data used in this study was obtained from the EEG databank from the Biomedical Engineering Laboratory of Tampere University of Technology (TUT), Finland. The measurements were carried out at the research laboratory of the Biomedical Engineering department of TUT at FinnMedi, Tampere, Finland. The EEG data used was the N170 component of VEP for three subjects S1, S2 and S3 under three different stimuli – fearful face, neutral face and control object. The three subjects had age range from 18 to 28 years and had no neurological history. It was a 256-channel data, with sampling rate of 1000 Hz and had 40 epochs. The EEGLAB [20] toolbox in MATLAB was used to average the potential data. The time period was from -100 milliseconds to 500 milliseconds. The signal at 170 ms was extracted from the averaged data for all the three subjects and was used to compute the inverse solution. The gain of the amplifier was set to 5000 and the signal was filtered with band pass filter of frequencies between 0.1 Hz to 200 Hz.

### Electrode locations

The realistic electrode locations obtained were projected on the scalp surface of the FDM model. The scalp surface nodes were extracted from the FDM model as the nodes lying at the interface of skin and empty space. The co-ordinates of the points obtained from the Polhemus system was transformed into a new orthogonal co-ordinate system defined using the image dataset. The nasion and the pre-auricular points were on the *x-y* plane. The nasion was along the *x*-axis and the pre-auricular points were on the *y-*axis. The realistic electrode locations were first projected on a unit sphere. This sphere was placed at the origin of the new orthogonal co-ordinate system and the electrode locations were projected back onto the scalp surface.

### Discrete Picard condition

Determination of the decay rate of Fourier co-efficients with respect to the singular values is important to investigate the properties of regularised solution. The discrete Picard condition states that, if the Fourier co-efficients on an average, decay faster than the singular values of the transfer matrix, then the regularised solution will have the same regularity properties as the exact solution [21]. To investigate this, we obtained the discrete Picard plot for the measurement data concerning all the three stimuli for subjects S1, S2 and S3. The regularisation toolbox was used to obtain Picard plots[25].

### Estimation of the cortical potential distribution

The forward transfer matrix **A**, vector of measured scalp potential **b** and the solution vector **x** are related by,

Ax = b (1)

 To obtain the inverse solution, zero-order Tikhonov regularisation was applied [22]. It is given by,

X = (A^T^ A + αI)^–1^A^T^ b (2)

where α is the regularisation parameter. In this paper, two methods: L-curve and GCV are employed to find this parameter. In L-curve method, the α is given as the corner of a curve obtained by plotting the solution norm versus the residual norm [23]. In GCV method, the value of α for which the GCV curve attains its minimum is considered to be the optimal value [24]. The GCV function is given by,

 (3)

where the factor denotes the matrix that maps the known vector, **b** onto the regularized solution. The regularisation toolbox [25] was used to compute the parameter and apply regularisation technique.

### Comparison of cortical potential distribution

The cortical potential distribution obtained using GCV and L-curve method can be compared by computing what is known as the MAG index and RDM index [34].

The MAG and RDM index are given as,

 (4)

 (5)

where, *n* is the number of source areas, X_GCVi_ and X_Li_ are the cortical potential values for *i^th^* source area computed using GCV and L-curve methods respectively.

The MAG index is used to compare the magnitude of potential values, where as the RDM index gives the fitting quality of spatial distribution of potential. In case of perfect fitting, the MAG would be 1 and RDM would be 0 [34].

## Results

### The discrete Picard plot

**Figure 2:**
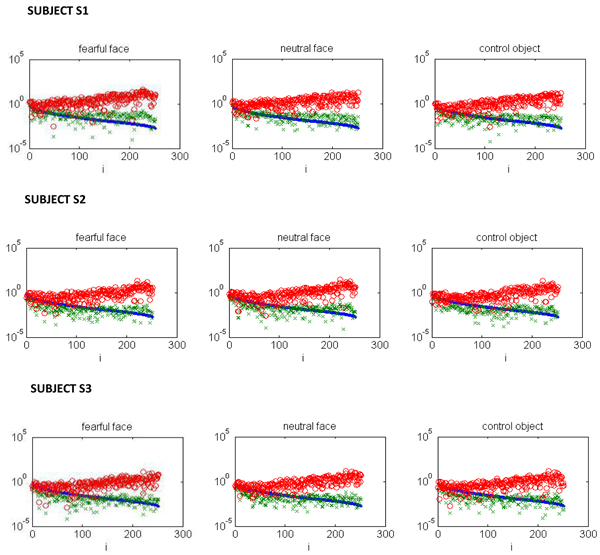
The Picard plot for fearful face, neutral face and control object stimuli (from left to right) . The blue dots are the singular values, the Fourier coefficients are shown in green and red circles are the red circles are Fourier coefficients divided by singular values.

The discrete Picard plots were obtained for subjects S1, S2 and S3, for three visual stimuli – fearful face, neutral face and control objects. These plots are shown in Figure 2. We have found that on an average, the rate of decay of Fourier co-efficients for the data concerning subject S1 was slower than the decay of singular values. For the data concerning subjects S2 and S3, the rate of decay of Fourier co-efficients on an average, was faster than the decay of singular values. This can be observed visually from Figure 2.

### The L-curve and GCV functional curve

**Figure 3:**
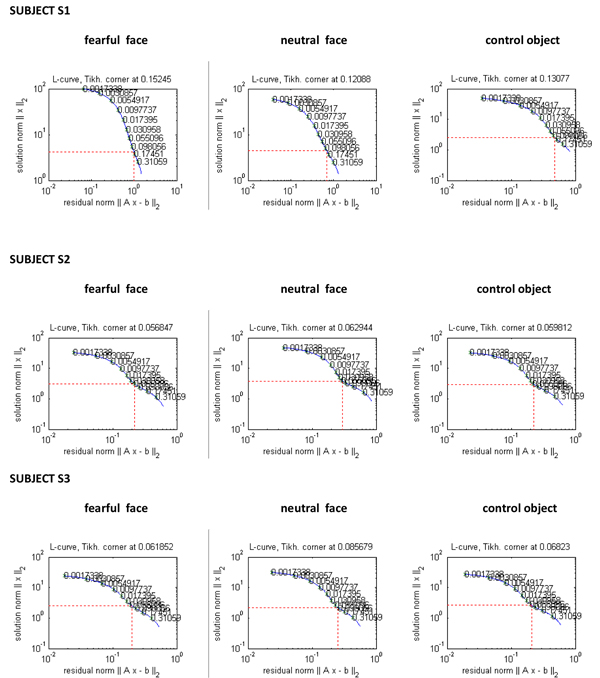
The L-curve for fearful face, neutral face and control object stimuli ( from left to right) for all the three subjects. The residual norm is on *x*-axis and solution norm on the *y*-axis.

**Figure 4:**
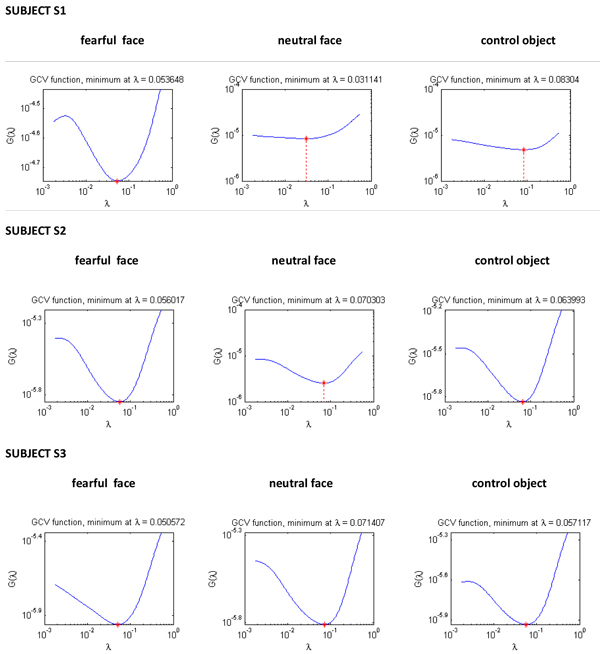
The GCV functional for fearful face, neutral face and control object stimuli ( from left to right) for all the three subjects. The regularisation parameter is on *x*-axis and GCV functional on the *y*-axis.

We computed the regularisation parameter for three subjects using L-curve method. The L-curve for subjects S1, S2 and S3 can be seen in Figure 3. We can see that, in case of subject S1 the shape of L-curve is not its characteristic ‘L’ and it breaks down displaying non-convergence. For subjects S2 and S3, the L-curve is convergent and gives a corner, which is discernible. Figure 4 shows the GCV functional for S1, S2 and S3. We can see that the GCV functional gives a discernible minimum for all the subjects. Although in case of neutral faces and control object stimuli for subject S1, the GCV curve looks somewhat flat deviating slightly from its characteristic shape, but still gives a well defined minimum value without breaking down.

Table 2 gives the list of values of regularization parameter for all the stimuli along with MAG and RDM index for S1, S2 and S3. There is a good correspondence between the values of regularization parameter between L-curve method and GCV method in case of S2 and S3, where as in case of S1 the difference is appreciable.

**Table 2:** Regularisation Parameter, MAG, RDM

Subject	Stimuli	GCV	L-curve	MAG	RDM
S1	fearful face	0.053647904825390	0.152452747854906	2.056	0.324
S1	neutral face	0.031140597094695	0.120884943960203	2.294	0.480
S1	control object	0.083040289002053	0.130773447052198	1.255	0.063
S2	fearful face	0.056017051286285	0.056846917686050	1.005	5.03e-5
S2	neutral face	0.070302890955651	0.062943521788770	0.967	2.17e-4
S2	control object	0.063992953233160	0.059812187027973	0.977	9.16e-4
S3	fearful face	0.050572034666208	0.061852135011805	1.071	0.0072
S3	neutral face	0.071407469554866	0.085678918300168	1.078	0.0084
S3	control object	0.057116631606758	0.068229559915199	1.069	0.0077

Comparison between the regularisation parameters, MAG index and RDM index between the cortical potential distributions obtained using L-curve and GCV method for all the three subjects. A value of MAG close to 1 and RDM close to 0 indicates a good fit. For S2 and S3 the fit is good, where as for S1 there is appreciable deviation.

### Cortical potential distribution for N170

**Figure 5:**
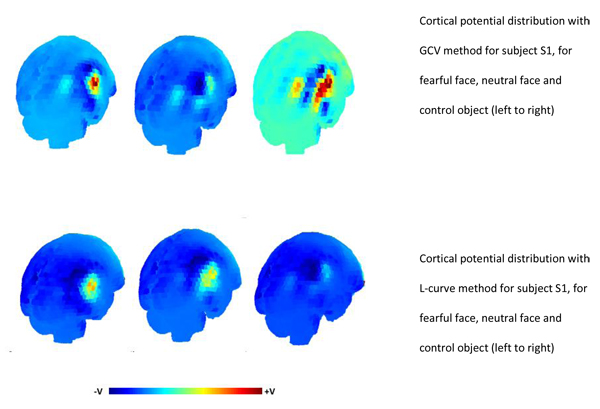
Cortical potential distribution for subject S1 using GCV method (Top panel) and L-curve (bottom panel). The solutions obtained are for fearful faces, neutral faces and control objects (left to right).

**Figure 6:**
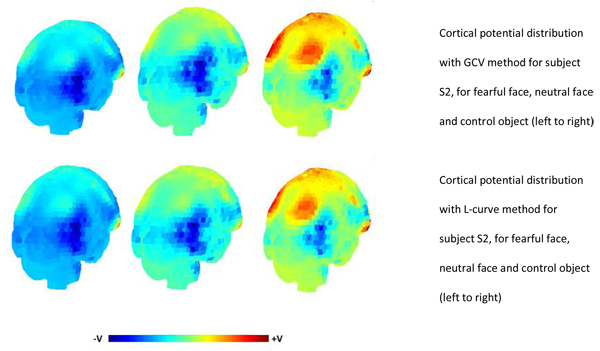
Cortical potential distribution for subject S2 using GCV method (Top panel) and L-curve (bottom panel). The solutions obtained are for fearful faces, neutral faces and control objects (left to right).

**Figure 7:**
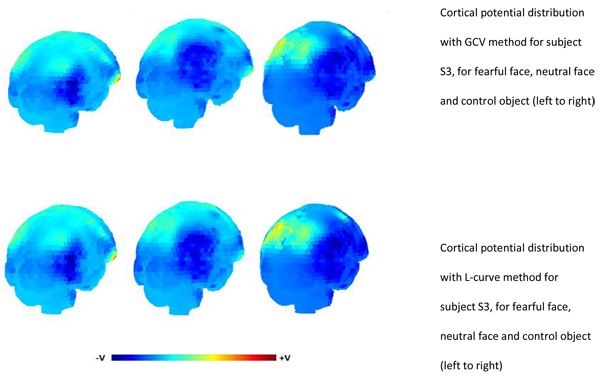
Cortical potential distribution for subject S3 using GCV method (Top panel) and L-curve (bottom panel). The solutions obtained are for fearful faces, neutral faces and control objects (left to right).

The cortical potential distribution obtained using GCV method and L-curve to select the regularisation parameter for all the three subjects are shown from Figures 5, 6, 7. The results indicate that in case of S2 and S3, there is an area of maximum negative potential on the right hemisphere of the cortex at the occipito-temporal part. This was a common observation from the cortical potential distribution obtained using L-curve and GCV method for the three stimuli. For subject S1, the results obtained from these two methods and were different. The potential distribution obtained using GCV method for S1 showed areas of negative potential around the occipito temporal cortex and small areas of positive potential in the temporal parts. The potential distribution from L-curve showed characteristics of over-smoothing and blurring. A large patch of negative potential was observed over the occipital area for S1. This is probably due to the high value of regularisation parameter obtained for this subject that resulted in over-smoothed solution. The MAG and RDM index for the potential distributions obtained using both the methods are also shown in Table 2. These values confirm that, the cortical potential distribution obtained using L-curve and GCV method for subjects S2 and S3 has a good fitting with each other, since the MAG index value is close to 1 and RDM index value is close to 0. In case of subject S1, the MAG and RDM index values deviate quite much from 1 and 0.

## Discussion

The selection of regularisation parameter is the key issue in the implementation of regularisation methods for solving ill posed problems. It is the regularisation parameter that controls the smoothness of the solution and tuning of this parameter is essential in arriving at a meaningful and reliable solution. Two widely used and popular methods, L-curve and GCV method are compared in this study.

The L-curve method has emerged as a popular method over the past few years [23]. The L-curve method generally produces a sharp, convex corner shaped as ‘L’. However, this always may not be the case. The L-curve might breakdown due to the dominance of correlated geometry noise over the Gaussian measurement noise and such instances have been reported in literature [26] or due to the violation of the discrete Picard condition [27]. The GCV method is based on successively leaving out elements of measurement vector and computing the regularised estimate of the solution vector from the reduced dataset. It predicts the left out response from this newly estimated model. Unlike L-curve, under the circumstances of violation of discrete Picard condition, GCV method has performed well [28,29].

The EEG potential data acquired for subject S1 was not of good quality as the electrodes near the right ear had high impedances. It was observed that, the discrete Picard condition was not satisfied in case of S1 as seen from Figure 2, which may have lead to the poor performance and breakdown of L-curve. Such instances of breakdown have been reported in the past [27,30].

In this study, we observed that in the L-curve for data concerning S1, the residual norm was monotonically increasing with no considerable change in the semi-norm of the solution as evident from Figure 3. The regularised solution obtained using the regularisation parameter from L-curve for S1 was over-smoothed and blurred. This can be seen in Figure 5. The GCV method performed relatively well in case of S1, in spite of the violation of discrete Picard condition. The GCV curve gave a sharp minimum for the fearful face stimuli. In case of neutral face and control object, the GCV method still performs well maintaining its characteristic shape and giving a well defined minimum. In case of S2 and S3, where the data was of good quality and the discrete Picard Condition was satisfied, both the methods gave comparable regularisation parameter, which in fact were not very different from each other.

We also computed the cortical potential distribution for the N170 component using the regularisation parameter obtained from this study for all the three subjects. There have been studies in the past regarding the source localisation of the N170 component to faces and objects [31-33], but none have focussed on the cortical potential distribution. These studies have demonstrated that the probable sources for facial perception are located mainly in the fusiform gyrus, posterior superior temporal sulcus region, predominantly on the right hemisphere. In this study, our aim was not to localise the sources, but to compute the equivalent of scalp potential distribution over a closed surface, defined as cortex. Though such a distribution gives no information about the underlying complex set of sources, but it can be helpful in providing hints about the sources. From the cortical potential distribution computed from this study for the N170 component of the ERP, we can see that the maximal negative potential occurs around the occipital and temporal parts of the cortex. The fusiform face area, which is thought to be responsible for facial recognition is located on the fusiform gyrus and is larger in the right hemisphere, though there is slight variation from person to person. The cortical potential distribution computed in this study seemed consistent with this fact by displaying areas of high negative potential on the right hemisphere of cortex and such a potential distribution probably may give significant hints about the putative sources. To our knowledge, there has been no study that has aimed to analyse the potential distribution over the cortex for the N170 component. Though many studies have aimed at source localization, computation of potential distribution over the cortex is a lesser explored research area.

In future work, we plan to use subject – specific models and automatically digitise the electrode locations. Also, an accurate representation of cortex is needed including all the sulcus and gyri, to get a better spatial detail for the potential distribution.

## Conclusions

The GCV method is more robust method than the L-curve method in finding the regularisation parameter even when there is noise dominance and discrete Picard condition is not satisfied. Cortical potential imaging technique is a feasible method to investigate the cortical potential distribution for N170 component of the VEP data.

## Competing interests

The authors declare that they have no competing interests.

## Authors' contributions

The first author has designed the studies, implemented the methods, prepared the results and wrote the manuscript. The other three authors have guided and reviewed the paper.
